# Dual Delivery of Hepatocyte and Vascular Endothelial Growth Factors via a Protease-Degradable Hydrogel Improves Cardiac Function in Rats

**DOI:** 10.1371/journal.pone.0050980

**Published:** 2012-11-30

**Authors:** Apoorva S. Salimath, Edward A. Phelps, Archana V. Boopathy, Pao-lin Che, Milton Brown, Andrés J. García, Michael E. Davis

**Affiliations:** 1 Parker H. Petit Institute for Bioengineering and Bioscience, Georgia Institute of Technology, Atlanta, Georgia, United States of America; 2 George W. Woodruff School of Mechanical Engineering, Georgia Institute of Technology, Atlanta, Georgia, United States of America; 3 Wallace H. Coulter Department of Biomedical Engineering, Emory University and Georgia Institute of Technology, Atlanta, Georgia, United States of America; 4 Division of Cardiology, Emory University School of Medicine, Atlanta, Georgia, United States of America; Institute of Clinical Medicine, National Cheng Kung University, Taiwan

## Abstract

Acute myocardial infarction (MI) caused by ischemia and reperfusion (IR) is the most common cause of cardiac dysfunction due to local cell death and a temporally regulated inflammatory response. Current therapeutics are limited by delivery vehicles that do not address spatial and temporal aspects of healing. The aim of this study was to engineer biotherapeutic delivery materials to harness endogenous cell repair to enhance myocardial repair and function. We have previously engineered poly(ethylene glycol) (PEG)-based hydrogels to present cell adhesive motifs and deliver VEGF to promote vascularization *in vivo.* In the current study, bioactive hydrogels with a protease-degradable crosslinker were loaded with hepatocyte and vascular endothelial growth factors (HGF and VEGF, respectively) and delivered to the infarcted myocardium of rats. Release of both growth factors was accelerated in the presence of collagenase due to hydrogel degradation. When delivered to the border zones following ischemia-reperfusion injury, there was no acute effect on cardiac function as measured by echocardiography. Over time there was a significant increase in angiogenesis, stem cell recruitment, and a decrease in fibrosis in the dual growth factor delivery group that was significant compared with single growth factor therapy. This led to an improvement in chronic function as measured by both invasive hemodynamics and echocardiography. These data demonstrate that dual growth factor release of HGF and VEGF from a bioactive hydrogel has the capacity to significantly improve cardiac remodeling and function following IR injury.

## Introduction

Cardiovascular disease is the leading cause of death in the United States, with estimates indicating 1 death every 39 seconds. Coronary heart disease, specifically myocardial infarctions (MI), accounts for 1 of every 6 deaths in the Unites States with over 1 million new, recurrent, and silent MI annually [1]. Following an occlusive coronary event, patients are treated with percutaneous coronary intervention to clear the affected vessel and restore blood flow [2]. While life saving, the ischemia and subsequent reperfusion (IR) induces massive regional necrosis and apoptosis, with billions of myocytes being lost over the first few days [3], [4], [5]. These lost cells are not replaced and a non-contractile scar is laid down, which will eventually lead to heart failure. Currently, the only definitive cure for heart failure is cardiac transplantation, and the number of donor hearts needed vastly outweighs availability. Thus, new therapies to treat this progressive disease and improve cardiac function are greatly needed.

Because the loss of tissue is highly localized, and the endogenous response is not sufficient for repair, recent efforts have focused on replacement of the lost cells using a variety of treatment options [6]. In recent years, many clinical studies have been initiated to deliver localized therapy in the form of various cell types for reconstitution of the myocardium. However, there is much debate on the optimal cell type, whether or not stem cells can differentiate into functional myocardium and the long-term effects of these non-myocytes [7], [8]. In addition to exogenous cell delivery, paracrine effects arising from delivery of angiogenic factors and other biochemical agents suggest that the myocardium retains the ability to remodel and heal [9]. Understandably, there has been tremendous focus on both growth factor- and gene therapy-based therapeutics. Whereas a source of great promise, direct growth factor delivery to the myocardium will most likely be inefficient as several studies have noted that many of these proteins are carried away in the highly vascularized cardiac tissue thereby limiting the effective tissue dose [10], [11], [12]. Gene therapy, while providing an excellent analytical tool, has not met with enthusiasm clinically, mostly due to the inability to quantify delivery and nonspecific targeting *in vivo*.

The need for spatiotemporal control to treat cardiac dysfunction is clear because ischemia/reperfusion (IR) injury is progressive with a distinct temporal and spatial response demanding distinct, controlled intervention [4]. Each of these processes is controlled by distinct mechanisms, and data suggest that therapeutics which may help at one phase may have no impact, or negative effects, at later time points. For example, small systemic doses of vascular endothelial growth factor (VEGF) improve regional blood flow following infarction; however, this did not lead to an improvement in cardiac function [13], [14]. However, large systemic doses of VEGF induce unfavorable side effects including hemangiomas, diabetic retinopathy, atherosclerosis, and rheumatoid arthritis [15]. Thus, there is a compelling need for temporal and spatial control of therapeutic delivery as one therapeutic intervention is unlikely to be optimal for all phases of post-MI healing.

In this study, a bioactive, protease-degradable polyethylene glycol (PEG)-based hydrogel was used to deliver hepatocyte growth factor (HGF) and VEGF following acute myocardial infarction. The ability of the growth factor-laden hydrogels to improve angiogenesis, fibrosis, and progenitor cell infiltration was examined at chronic time points and functional parameters determined using clinical echocardiography and invasive hemodynamics.

## Materials and Methods

### Generation of PEG-hydrogel

**Figure 1:**
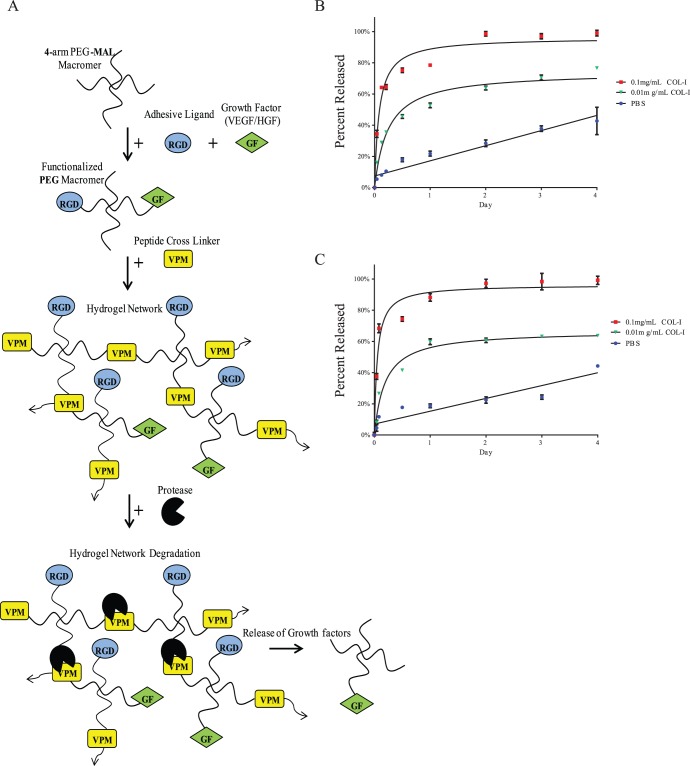
Hydrogel design and release studies. A) PEG-maleimide (PEG-MAL) 4-arm macromers were pre-functionalized with RGD adhesion peptide and growth factors HGF and/or VEGF. The precursor molecules were cross-linked into a hydrogel by addition of cysteine-flanked protease-degradable peptide sequence GCRDVPMSMRGGDRCG (VPM). B) Fluorescently tagged VEGF and C) HGF released from PEG-MAL hydrogel treated with either PBS or 2 doses of collagenase Type 1 as measured by fluorescent plate reader sampling of incubation media. One hundred percent of loaded VEGF and HGF were released by day four after collagenase treatment while lower doses of collagenase and PBS released up to 65% and 40%, respectively. Data are mean ± SEM from 6 samples per condition. Hyperbolic and linear curve fits indicate different release profiles behaviors for collagenase vs. PBS treatment.

Protease-degradable PEG-based hydrogels were prepared as previously described [16]. PEG-maleimide (PEG-MAL) 4-arm macromers (20 kDa, >95% maleimide functionalization; Laysan Bio) were pre-functionalized with GRGDSPC (RGD) adhesion peptide and growth factors (1% v/v) HGF and/or VEGF in 2.0 mM triethanolamine (TEA) solution. PEG polymer density used was 4% w/v and RGD density was 2.0 mM. The precursor molecules were cross-linked into a hydrogel by addition of a cysteine-flanked protease-degradable peptide sequence GCRDVPMSMRGGDRCG (VPM) [17], [18] (Fig. 1A). The final concentration of growth factors delivered was 1 µg VEGF (Gibco) and 1 µg HGF (US Biologicals) per 100 µl hydrogel.

### In vitro Release Study

HGF and VEGF were tagged with Alexa Fluor 488, Carboxylic Acid, Succinimidyl Ester, mixed isomers (Invitrogen), proteins were incubated at 0.1 mg/mL at a 1∶50 protein to dye molar ratio in sodium bicarbonate buffer for 2 hours at 37°C, and purified into PBS by four passes through a desalting spin column (Zeba Spin Desalting Column, Pierce, 7K MWCO). Labeled protein was snap frozen in liquid N_2_ and stored at −80°C. Protein samples were confirmed to be labeled with respective Alexa Fluor tag, free from excess dye, by SDS-PAGE imaged on a BioRad system under Trans UV light.

To track the release of HGF and VEGF from PEG-MAL hydrogels, Alexa Fluor-tagged growth factors were pre-incubated with 4-arm PEG-MAL macromer for 1 hour and incorporated into PEG-MAL hydrogels by crosslinking remaining unreacted MAL groups with VPM protease degradable peptide. Gels were incubated in PBS alone or with 0.1 mg/mL or 0.01 mg/mL collagenase Type 1 (Worthington Biochemical) for 4 days at 37°C. Individual hydrogel volumes were 50 µL and 5 uL of tagged growth factor was added such that final concentration in the gel was equal to 0.02 mg/mL. Samples of incubation media were taken every few hours for day 1 and then every 24 hours and read using a standard fluorescence plate reader (Perkin Elmer). Percent released was calculated as fraction of the dye released/maximum loaded as compared to a standard dilution for each tagged growth factor.

### Animal Studies

A randomized and blinded study was conducted using adult Sprague-Dawley rats (Charles River) weighing 250 g. Rats were randomly assigned to treatment groups (n = 7–10) using a random generator and the surgeon was only given letter codes to identify groups. While one group was subjected to sham surgery, the other eight groups received ischemia/reperfusion (IR) surgery (30 min. coronary artery ligation followed by reperfusion), with or without the injection of 100 µl (containing 1 µg) of the following: free VEGF, free HGF, free VEGF+HGF, PEG only, PEG/VEGF, PEG/HGF, or PEG/VEGF+HGF. Injections were made into the perimeter of cyanotic ischemic zone (3 locations) through a 30-gauge needle immediately after reperfusion. The animals were allowed to recover and functional assessments were made at later time points. The animals were sacrificed 21 days following surgery and the hearts were fixed in 4% paraformaldehyde, dehydrated, embedded in paraffin, and sectioned for immunohistochemical analysis.

This investigation conformed with the *Guide for the Care and Use of Laboratory Animals* published by the US National Institutes of Health (NIH Publication No. 85-23, revised 1996) and all animal studies were approved by Emory University Institutional Animal Care and Use Committee (Animal Welfare Assurance #A3180-01).

### Echocardiography

Anesthetized rats were subjected to echocardiography at 7 and 21 days following IR surgery. Short axis values of left ventricular end systolic (ES) and end diastolic (ED) dimension were obtained using Acuson Sequoia 512 echocardiography workstation with 14 MHz transducer. An average of 3 consecutive cardiac cycles was used for each measurement and was performed three times in an investigator-blinded manner. Fractional shortening was calculated as (end-diastolic diameter – end-systolic diameter)/end-diastolic diameter and expressed as a percentage.

### Invasive Hemodynamics

Following echocardiography at 21 days, animals were subjected to invasive hemodynamic measurements using a Millar MPVS Pressure-Volume system and Power Lab software package. Following intubation, a 2-French catheter was inserted into the right jugular vein and advanced to the right ventricle for pressure measurements. After the catheter was removed from the right ventricle, a polyethylene catheter was inserted into the right jugular vein for the subsequent saline volume challenge. The pressure-volume catheter was then inserted into the right carotid artery and advanced to the left ventricle. After stabilization, baseline left ventricular pressure-volume loops were recorded. To change preload, the inferior vena cava was transiently compressed (<30 seconds) through an incision in the upper abdomen. Pressure-volume measurements were recorded for at least 10 cardiac cycles and data averaged to get mean values for each animal. Data extracted include +dP/dT, -dP/dT, left-ventricular end systolic and diastolic pressure and volume.

### Immunohistochemistry

Paraffin-embedded tissue was sectioned in to 5 mm sections and adhered to a glass slide. Following rehydration, tissue sections were probed with FITC-labeled isolectin B4 (Molecular Probes) and imaged using fluorescence microscopy. Additionally, tissue sections were probed against c-kit using a commercially available antibody (Santa Cruz H-300) and a fluorescently labeled secondary antibody (Molecular Probes). To quantify immunohistochemistry, 5 non-serial sections from each animal were analyzed by a blinded investigator and expressed as either vessels/mm^2^ (for isolectin) or cells/mm^2^ (for c-kit).

Collagen deposition was determined by picrosirius red (Sigma) staining as previously described [19]. Briefly, 5 µm tissue sections were stained with Sirius Red and imaged at 2.5× using a light microscope. Overlapping images were taken and then stitched together using Adobe Photoshop to generate an image of the entire heart. Total collagen area (red staining) was normalized to total left ventricular area in 3 separate sections per animal using ImageJ.

### Migration

Cardiac progenitor cells (CPCs) were isolated from adult rat hearts as described using anti-c-kit coated magnetic beads prior to clonal expansion [20]. The CPCs were characterized for expression of c-kit and only clones with >90% expression of c-kit and >80% expression of the cardiac transcription factors nkx2-5 and gata-4 were used in this study. CPCs were trypsinized and incubated with 5-chloromethylfluorescein diacetate (CMFDA; Molecular Probes) for 30 minutes at 37°C. Four percent PEG hydrogels of 50 µL size containing 10 ng of VEGF, HGF or VEGF+HGF were generated as described above. The gels were laid flat into 8 well Labtek chamber slides with glass bottom (Nunc) for ease of imaging. The hydrogels were swollen in 100 µL PBS with Penicillin/Streptomycin for one hour at which point they completely covered the bottom of the well. CMFDA labeled CPCs (50,000 ) were seeded on top of the flat hydrogel surface. After 48 hours, the extent of CPC migration into the hydrogel was visualized on a Nikon-C1 laser scanning confocal microscope with a 20× air objective. Five z-stack projections through a 90–450 µm thick section of the swollen hydrogel were rendered. The thickness of the imaged section was determined by the minimal thickness required to observe maximum cellular migration to reduce image capture and processing time. The maximum distance migrated by the cells into the hydrogel was calculated using ImageJ.

### Statistics

All statistical analyses were performed using Graphpad Prism 5 software as described in the figure legends. P values of less than 0.05 were considered significant.

## Results

### Growth Factor Release from Hydrogels

Bioactive hydrogels were synthesized as depicted in Figure 1A. To determine release of VEGF (Figure 1B) and HGF (Figure 1C) in vitro, fluorescently-labeled growth factors were chemically incorporated into PEG-MAL hydrogels. Modification of growth factors by PEG-MAL was verified by molecular weight increases of the protein band on SDS-PAGE. Hydrogels subjected to higher collagenase treatment were observed to degrade within the first 48 hours after treatment while hydrogels in PBS remained intact. Samples incubated with increasing levels of collagenase released loaded growth factor rapidly coinciding with gel degradation. PBS treated samples released protein much more slowly. These results show the rate of release of growth factors is dependent upon protease-mediated degradation of the hydrogel due to the protease-degradable crosslinking peptides.

### Angiogenesis

**Figure 2:**
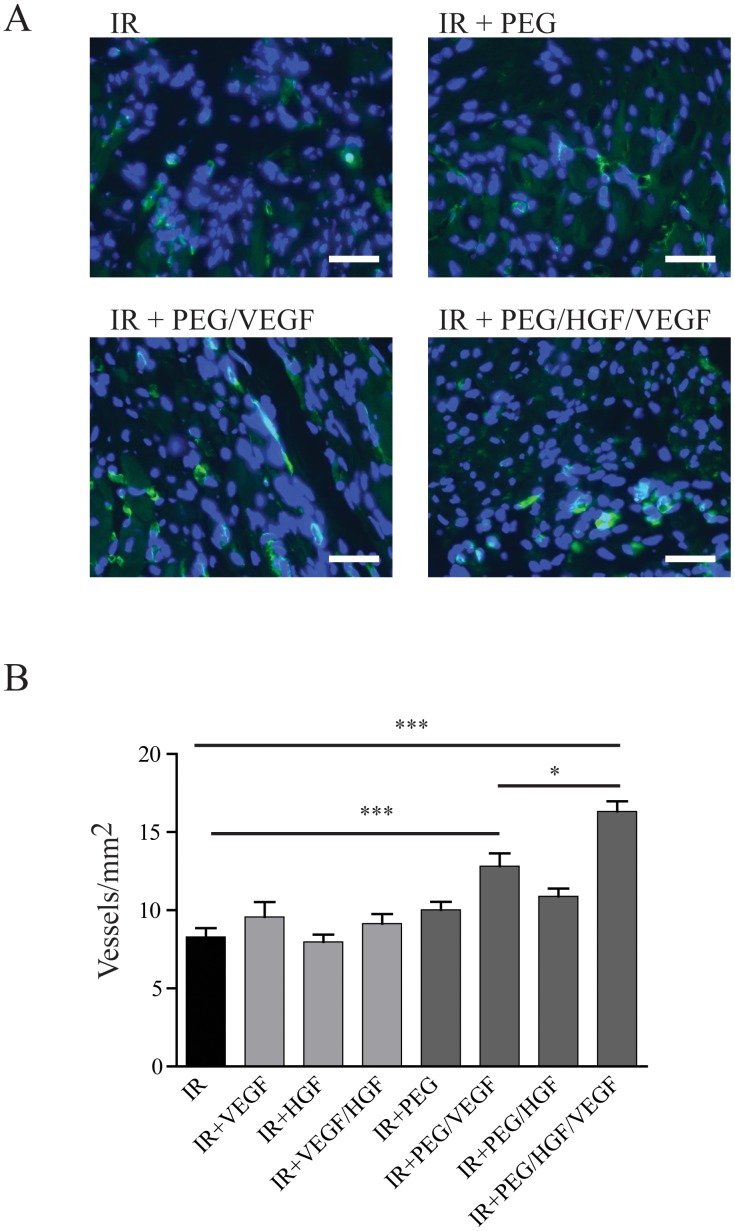
Angiogenesis following ischemia-reperfusion in treated rats. At 21 days following infarction, vessels were quantified by staining with isolectin-b4 conjugated to FITC. (A) Representative images (green = isolectin; blue = DAPI; scale bar = 30 µm) of rats subjected to IR, IR + PEG, IR+PEG/VEGF, or IR + PEG/HGF/VEGF. B) Grouped data (mean ± SEM; n≥7 per group) demonstrating a significant increase in vessels/mm^2^ in the PEG/VEGF rats compared with IR alone, and in the PEG/HGF/VEGF rats compared with either PEG/VEGF or IR alone (***p<0.001; *p<0.05; ANOVA followed by Student-Newman-Keuls post-test). PEG = polyethyleneglycol, VEGF = vascular endothelial growth factor, HGF = hepatocyte growth factor, IR = ischemia reperfusion.

Rats were subjected to ischemia-reperfusion (IR) surgery and free growth factors or growth factors embedded in protease-degradable hydrogels were injected in to the borderzones. To determine levels of angiogenesis, sections were probed with a FITC-labeled isolectin conjugate and vessels were counted 21 days following infarction. In untreated animals, 8.3±0.5 vessels/mm^2^ were counted in the infarcted area (Figure 2A top left). Infarcts treated with free VEGF, PEG hydrogel alone, or HGF-loaded PEG hydrogels (PEG/HGF) had equivalent levels of angiogenesis as the untreated control. Treatment of animals with VEGF-loaded PEG hydrogels (PEG/VEGF) significantly increased vessel density in the infarct area (12.8±0.8 vessels/mm^2^; p<0.01 vs. IR alone; Figure 2A bottom left). Interestingly, PEG hydrogels loaded with VEGF and HGF (PEG/VEGF+HGF) further increased vessel density over PEG/VEGF (16.3±0.6 vessels/mm^2^; p<0.05 vs. PEG/VEGF, p<0.001 vs. IR alone; Figure 2A bottom right). These grouped data (Figure 2B) demonstrate that PEG/VEGF is able to induce significant angiogenesis following MI, and that the presence of HGF along with VEGF induces a synergistic response.

### Fibrosis

**Figure 3:**
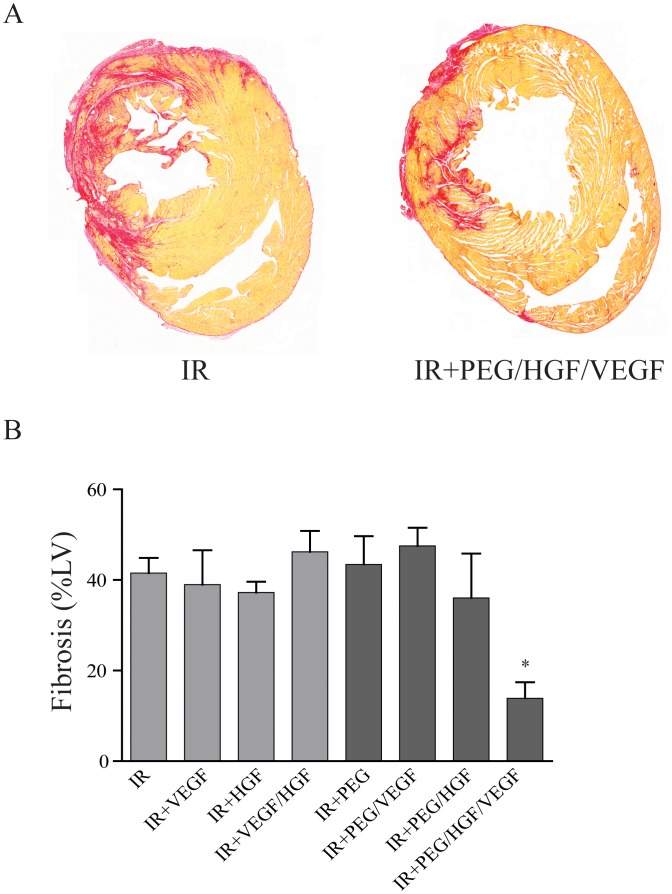
Fibrosis following ischemia-reperfusion in treated rats. At 21 days following infarction, fibrosis was measured by staining for collagen using picrosirius red (red stain, images taken at 20× magnification). A) Shows 2 representative stitched heart sections used for quantification (IR alone and IR + PEG/VEGF/HGF. B) Grouped data (mean ± SEM; n≥7 per group) demonstrating a significant decrease in fibrotic area in PEG/HGF/VEGF rats compared with IR alone (*p<0.05; ANOVA followed by Student-Newman-Keuls post-test). PEG = polyethyleneglycol, VEGF = vascular endothelial growth factor, HGF = hepatocyte growth factor, IR = ischemia reperfusion, LV = left ventricle.

To determine levels of fibrosis following MI, tissue sections were stained with picrosirius red 21 days following IR and fibrotic area was normalized to total LV area. Animals receiving no treatment had a fibrotic area of 41.5±3.3% of total LV area (Figure 3A left). There was no significant decrease in groups treated with free growth factors or empty hydrogel. Only PEG/VEGF+HGF-treated animals demonstrated a significant decrease in fibrosis compared with IR alone (13.9±3.5%; p<0.05; Figure 3A right). The grouped data in Figure 3B clearly demonstrates that only controlled release of both growth factors from the PEG hydrogel inhibited collagen deposition following IR.

### Progenitor Cell Isolation and Migration

**Figure 4:**
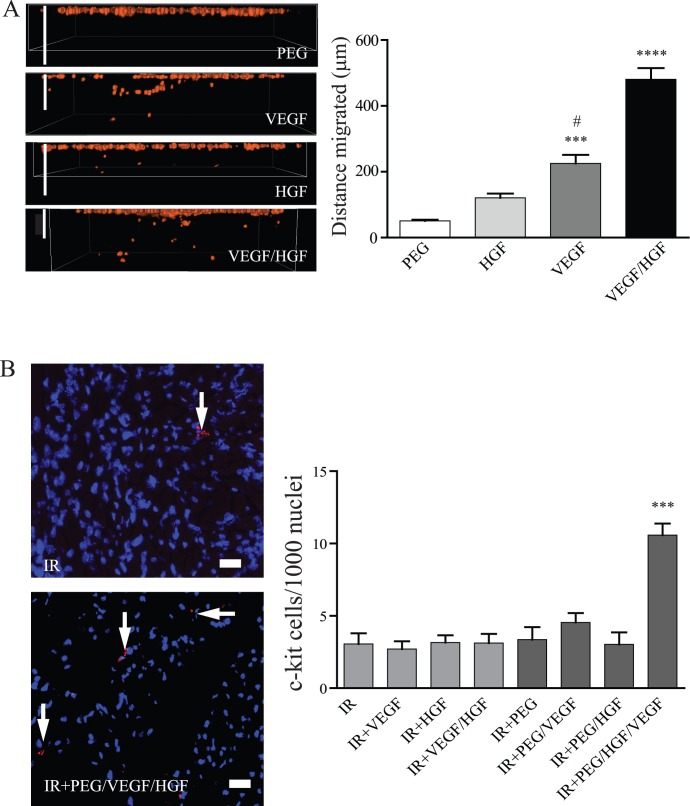
Progenitor cell recruitment. (A) Isolated, fluorescently-tagged cardiac progenitor cells (CPCs) were seeded on top of hydrogels containing nothing, VEGF, HGF, or HGF/VEGF and allowed to migrate for 48 hours. Gels were turned sideways and imaged using confocal microscopy and distance migrated was determined using ImageJ (scale bar = 100 µm). Data are mean ± SEM from 5 separate experiments and demonstrate a non-significant increase in migration in to HGF-loaded gels, but a significant increase in migration in to VEGF-loaded gels (***p<0.001 compared to PEG alone, #p<0.05 compared to HGF). There was a further increase in migration between the single treatment groups and HGF/VEGF (****p<0.0001 vs. all groups; ANOVA followed by Student-Newman-Keuls post-test). (B) Representative images of c-kit staining (red) in the infarct zone of rats with IR alone or treated with PEG/HGF/VEGF (blue = DAPI; scale bar = 30 µm). Grouped data (mean ± SEM; n≥7 per group) demonstrating a significant increase in c-kit^+^ cells in PEG/HGF/VEGF treated rats compared with IR alone (***p<0.001; ANOVA followed by Student-Newman-Keuls post-test). PEG = polyethyleneglycol, VEGF = vascular endothelial growth factor, HGF = hepatocyte growth factor, IR = ischemia reperfusion, RFU = relative fluorescent units.

To determine the effect of growth factor treatment on progenitor cell migration, *in vitro* migration assays were performed in addition to *in vivo* staining of progenitor cells. As shown in the representative images and grouped data in Figure 4A, c-kit^+^ cardiac progenitor cells demonstrated significant increases in migration in response to both HGF and VEGF *in vitro*. Examination of migration in to the hydrogel showed a significant increase in VEGF-loaded hydrogels, but not HGF-loaded hydrogels (p<0.001 VEGF vs. PEG alone; p<0.05 vs. HGF). There was a further increase in migration in to hydrogels loaded with both growth factors (p<0.0001 vs. VEGF, HGF, and PEG alone). Additionally, a similar trend was seen in migration in a 2-dimensional transwell system (data not shown). To determine levels of progenitor cells *in vivo*, sections were stained with an anti-c-kit antibody and positive cells were counted in relation to total cells. While all other treatments were similar to IR alone, there was a significant increase (p<0.001) in c-kit^+^ cells in animals treated with PEG/VEGF+HGF (IR = 3.1 c-kit^+^/1000 nuclei, PEG/VEGF+HGF = 10.6 c-kit^+^/1000 nuclei; Figure 4B&C).

### Echocardiographic Assessment of Function

**Figure 5:**
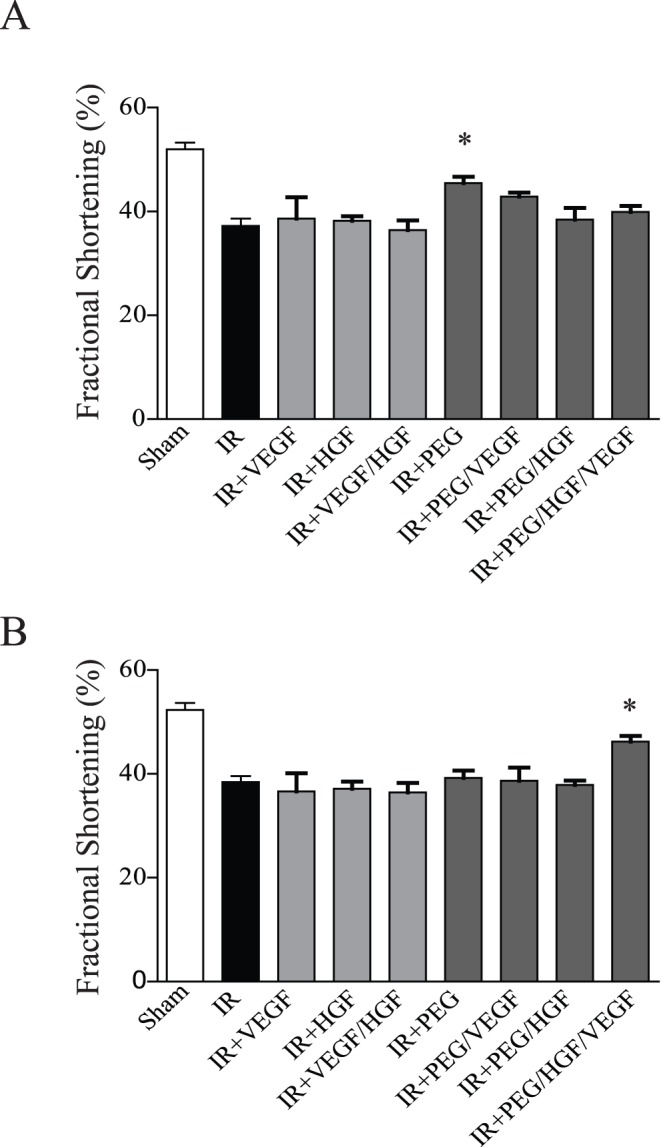
Echocardiographic measurement of function. Grouped data (mean ± SEM; n≥7 per group) from A) 7 days post-injury or B) 21 days post-injury. At 7 days there was a significant increase in function only in the empty PEG hydrogel treated rats. At 21 days, only the PEG/HGF/VEGF rats demonstrated a significant increase in function compared with untreated rats (*p<0.05; ANOVA followed by Student-Newman-Keuls post-test). PEG = polyethyleneglycol, VEGF = vascular endothelial growth factor, HGF = hepatocyte growth factor, IR = ischemia reperfusion.

To determine the effects of the various treatments on cardiac function, echocardiography was performed at 7 and 21 days following infarction. Data were obtained from short-axis images, quantified, and presented in Figures 5A&B, respectively. At day 7, there was a significant decrease in function in IR animals (p<0.001). Of all the treatment groups, only empty PEG hydrogels significantly improved function 7 days following IR (p<0.05 vs. IR alone). In contrast, 21 days following IR, empty PEG hydrogels were no different from IR alone. Interestingly, only PEG/VEGF+HGF-treated animals demonstrated a significant improvement in function (p<0.05 vs. IR alone). These data show that long-term improvements in function were only evident following combined growth factor delivery from PEG hydrogels.

### Invasive Hemodynamics

**Figure 6:**
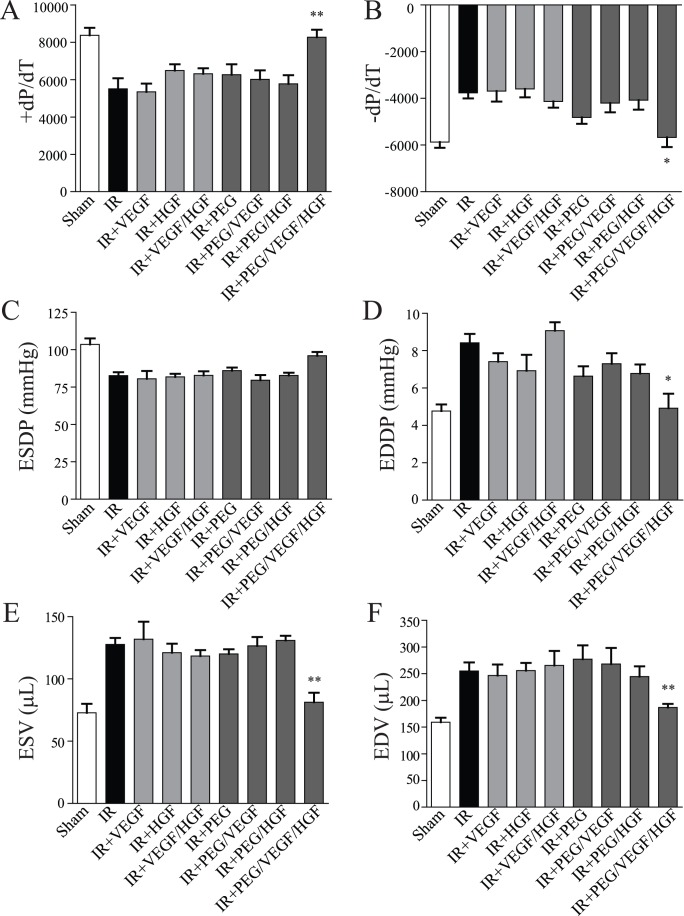
Invasive hemodynamic measurement of function. Grouped data (mean ± SEM; n≥7 per group) taken 21 days following injury by cardiac catheterization. A-F) Various measures of function demonstrate a significant improvement in all but 1 measurement (C, end-systolic developed pressure) in PEG/HGF/VEGF treated rats compared to IR alone (*p<0.05; **p<0.01; ANOVA followed by Student-Newman-Keuls post-test). PEG = polyethyleneglycol, VEGF = vascular endothelial growth factor, HGF = hepatocyte growth factor, IR = ischemia reperfusion, ESDP = end-systolic developed pressure, EDDP = end-diastolic developed pressure, ESV = end-systolic volume, EDV = end-diastolic volume, ±dP/dT = change in pressure over time.

To confirm echocardiographic assessments, invasive hemodynamics was performed to determine actual cardiac pressures and volumes 21 days following IR and summarized in Figure 6. Infarction induced a significant impairment in both positive and negative pressure changes over time (dP/dT; p<0.01 vs. sham). Of all the treatments, only PEG/VEGF+HGF-treated animals had significantly improved pressure changes (p<0.01 for +dP/dT, p<0.05 for –dP/dT vs. IR alone). In addition to pressure changes, significant improvements in left-ventricular end diastolic pressure (p<0.01) and both end systolic (p<0.05) and end diastolic volumes (p<0.01) were seen in PEG/VEGF+HGF-treated animals. Whereas there was a 20% improvement in left-ventricular end systolic pressure, it was not considered significantly different. Taken together, these data confirm echocardiographic measurements of cardiac function, demonstrating a significant improvement in cardiac function in animals receiving both growth factors delivered from a protease-degradable PEG hydrogel.

## Discussion

Ischemia-reperfusion injury (IR) results in excessive local cell death, and in many patients eventually leads to heart failure as the lost tissue is not replaced. While current treatments such as 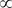-receptor antagonists and angiotensin receptor blockers are effective in improving contractility, there are few therapies that attempt to address the underlying mechanistic problems [2], [21]. While growth factor therapy holds great promise, the retention in the highly vascular myocardium is low and prevents sustained activation needed for adequate cellular responses [10]. Biomaterial-based approaches for sustained therapeutic delivery hold great promise as many have shown little toxicity in animal studies, and they can be tailored in many cases to address tissue needs.

In this report, we delivered a protease-degradable, bioactive PEG hydrogel loaded with VEGF and HGF to the infarct zone following IR for localized therapy. This material was chosen due to the low fouling properties of PEG, as well as excellent biocompatibility [22]. A recent publication with this novel material containing both RGD sequences to enhance cell adhesion, as well a dithiol protease-cleavable peptide cross-linker GCRDVPMSMRGGDRCG, demonstrate excellent cell compatibility as well as the ability to form *in situ* gels on tissue [16]. Timing of gel formation can be tuned by modifying the concentration of the buffering agent triethanolamine (TEA) in the reaction solution and thus proteins can be freshly added immediately prior to injection. The injected material is a liquid, yet gels rapidly in tissue. We examined VEGF and HGF release *in vitro* and show the hydrogel system is capable of controlled release due to protease-mediated degradation.

Preliminary studies demonstrated excellent *in situ* gel formation in the myocardium, similar to other hydrogels used for post-infarct drug delivery. In vitro release studies demonstrated a slow release profile in PBS that was accelerated by increasing levels of collagenase treatment. Given the amount of MMPs released following IR, especially collagenase [23], this on-demand release system is ideal for the post-infarcted heart, triggering release of growth factors when active remodeling is occurring. Interestingly, when function was measured at 7 days, only the empty hydrogel demonstrated a significant increase in function. While it is possible that the bioactive sequences aided in some manner, they are within the hydrogel and likely not diffusing away to enhance cellular function. Previous studies demonstrate that hydrogels themselves can acutely improve function due to mechanical support of the left ventricular (LV) wall [24], [25]. The bioactive PEG hydrogel has an elastic modulus of 0.5–1.0 kPa [16], within the range of most soft tissues and well below stiff substrates such as collagen that impede cardiac function [26], [27], [28]. While there was no effect of embedded growth factors at this early time point, it was not surprising as neither HGF nor VEGF plays a major role in cardiomyocyte survival, the major contributor of early regulation of cardiac function [6]. It is also possible that the growth factors were not released fast enough to diffuse to the injured cardiac tissue, although our in vitro results would suggest otherwise. In contrast to the day 7 data, at day 21 there was a significant improvement in cardiac function only in the group that received the bioactive hydrogel embedded with VEGF and HGF. In keeping with the acute studies, there was no effect of free growth factors, or single growth factor hydrogel treatments. This was determined both via 2D echocardiography and invasive hemodynamic measurements. Dual growth factor delivery from the bioactive hydrogel resulted in a significant decrease in end-systolic and diastolic volume, as well as improved contractile performance as evidenced by improvements in end-systolic and diastolic pressures.

To determine potential mechanisms for these enhancements, several areas were examined. Due to the documented effect of VEGF on angiogenesis [29], vessel number was assessed in the infarcted tissue. Consistent with previous findings, sustained VEGF delivery induced a significant increase in vessel number with no effect for free VEGF. Despite this increase in vessel number, this effect did not translate to increases in function. These data are supported by prior studies noting that angiogenesis alone may not be sufficient for long-term functional recovery [14], [30]. We saw no evidence of increased angiogenesis from HGF-loaded hydrogels, despite recent studies demonstrating the ability of HGF to induce angiogenesis following injury [31], [32], [33], [34]. Interestingly, the majority of these reports are using HGF gene therapy or various HGF-overexpressing cells [35], [36], suggesting perhaps long-term release of HGF is required for improved angiogenesis. However, long-term HGF production may have negative side-effects, and recent efforts have focused on using HGF gene expression that can be turned off via suicide gene system, indicating the need for temporal control of HGF release [37]. While our HGF release was not sustained enough to induce angiogenesis on its own, co-delivery of HGF with VEGF had a synergistic effect, significantly increasing angiogenesis over single hydrogel-growth factor delivery alone. This strategy of dual growth factor delivery has been used recently to co-deliver insulin-like growth factor-1 (IGF-1) with HGF to improve angiogenesis [38]. Additionally, both VEGF and IGF were identified as being potential mediators of the paracrine effects of stem cell therapy, specifically for improving angiogenesis [39], [40], [41]. Recent studies demonstrate that hydrogel-mediated co-delivery of VEGF and platelet-derived growth factor (PDGF) for angiogenesis is synergistic as compared with single growth factor therapy [42], [43]. Finally, both VEGF and HGF have been delivered synergistically using poly(lactic-co-glycolic) acid (PLGA) microspheres [44]. The authors made microspheres up to 100 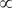
m in diameter and implanted them in the leg muscle of mice subjected to hindlimb ischemia. This interesting study found that dual factor-loaded microparticles themselves enhanced angiogenesis, but the main finding was that dual-delivery of HGF and VEGF enhanced endothelial progenitor cell-induced function and recovery compared to cells alone. While this is interesting in that it may be a future avenue to use our PEG hydrogel to deliver cells, previous reports from our laboratory demonstrate that PLGA microspheres are not optimal for post-infarction delivery due to uncontrolled release and potential to induce inflammation from acidic degradation products [19]. Taken together, our data clearly show that dual growth factor release from a bioactive hydrogel is superior to single factor release for improving angiogenesis, but that other combinations may also be effective and remains an area for potential future study.

Similar to angiogenesis, we also examined collagen content via picrosirius red staining and found the dual growth factor delivery to be significantly improved over single factor hydrogel-mediated or soluble growth factor delivery. The effect of VEGF on fibrosis is not clear, with many studies linking the ability of VEGF to reduce fibrosis on its ability to increase angiogenesis [45], [46]. While angiogenesis and fibrosis are clearly tied, there is potential for altered signaling in myocytes independent of angiogenesis. An early study demonstrated increased Akt-mediated signaling in animals treated with chronic HGF following infarction [47]. While Akt is well known for its role in cell survival, many studies link activation of Akt to decreases in collagen production and assembly, especially in fibroblasts [48]. While we cannot separate the specific cell signaling effects from the benefits of increased angiogenesis, it is worth noting that hydrogel-mediated VEGF delivery did not result in a decrease in fibrosis, indicating angiogenesis by itself may not be sufficient. Likewise, another explanation is that VEGF did not induce enough angiogenesis to improve fibrosis. We also examined progenitor cell infiltration by immunohistochemistry and found increased cell numbers only in the dual growth factor hydrogel group. Both VEGF [49] and HGF [50] are implicated in migration of c-kit^+^ cardiac progenitor cells (CPCs). Our in vitro data confirms that CPCs show increased migration in response to the synergistic combination of VEGF and HGF over single growth factor alone. Studies demonstrate that CPCs respond to gradients in growth factor concentrations in vivo, and the use of a biodegradable hydrogel may facilitate this. Additionally, the hydrogel used in this study also contains RGD ligands, which may facilitate CPC retention or binding [51]. It is also possible that increased angiogenesis allowed for better access for circulating and bone marrow-derived progenitors expressing c-kit. We do not know the source of c-kit^+^ cells in this study and could be an interesting area for future investigation. Finally, VEGF and HGF may also improve the survival and growth of migrated progenitor cells. While we did not see co-staining of c-kit with any vessels in our samples, it may be that c-kit expression was lost as the cells matured. Earlier studies demonstrated that bone-marrow-derived c-kit^+^ clusters had improved cardiac differentiation potential in vitro [52]. While we examined the slides for evidence of clusters in vivo, we were unable to detect any within the infarcted tissue. As the source of these cells was not determined in our studies, the role of cell clustering in the healing response seen is unknown, but remains an interesting area for future studies.

Our data clearly demonstrates the ability of a bioactive, degradable hydrogel to deliver growth factors in vivo. There are very few reports of PEG hydrogels used to improve post-MI remodeling and angiogenesis [53], [54]. The use of a hydrogel containing adhesive sequences, as well as degradable linkages is promising, as it allows for potential control over spatial and temporal aspects of healing and regeneration [16]. PEG hydrogels are promising in that they can be modified chemically, they have excellent biocompatibility, and do not bind proteins on their own. Thus, most parameters of drug delivery can be easily controlled. The processes following MI that contribute to the disease, inflammation, cell death, fibrosis, hypertrophy, all follow distinct time courses with varying locations [6]. Thus, it is unlikely that one factor will be optimal for all these events and multiple signals are needed. Our data demonstrate that dual growth factor delivery, sustained with bioactive PEG hydrogels is significantly better than free growth factors, or single-growth factor delivery attempts. While this seems rather intuitive, very few reports exist of dual growth factor delivery with biomaterials for MI. Thus the potential for creating even more complex delivery systems with control over growth factor release, cell migration, and scaffold degradation is quite promising.

### Conclusion

In this report, we demonstrate the first *in vivo* use of a recently described bioactive, protease-degradable PEG hydrogel capable of delivering multiple growth factors. The hydrogel was delivered via intramyocardial injection following infarction, and loaded either with VEGF, HGF, or both factors. Only the hydrogels loaded with both factors demonstrated functional improvements, with animals showing increased cardiac function by invasive hemodynamics and echocardiography. Our data demonstrate that dual growth factor release, specifically controlled release of both HGF and VEGF was far superior to single-factor delivery at inducing angiogenesis, inhibiting fibrosis, and stimulating migration of progenitor cells. These results may have clinical implications in the prevention of post-MI cardiac dysfunction and demonstrate the utility of synergistic, biomaterial-based growth factor delivery.
